# A82 DELIVERY OF AMBULATORY CARE DURING THE COVID-19 PANDEMIC IN THE DIVISION OF DIGESTIVE CARE & ENDOSCOPY, HALIFAX, NS

**DOI:** 10.1093/jcag/gwab049.081

**Published:** 2022-02-21

**Authors:** J Francheville, P Koto, K Peltekian

**Affiliations:** 1 Division of Digestive Care & Endoscopy, Dalhousie University, Halifax, NS, Canada; 2 Research Methods Unit, Nova Scotia Health Authority, Halifax, NS, Canada

## Abstract

**Background:**

The COVID-19 pandemic has placed the Canadian healthcare system under substantial strain requiring rapid and systemic changes to healthcare delivery in gastroenterology ambulatory care, including a shift to providing synchronous clinical visits virtually. It is important to describe and evaluate the impact of this care delivery change on patients, providers and the healthcare system in order to improve the quality of virtual care in the future.

**Aims:**

As part of a larger quality improvement initiative, the aim of this project was to better understand the health system impact of the shift from in-person to virtual care delivery in the Division of Digestive Care & Endoscopy in Halifax, NS.

**Methods:**

Using a before-and-after observational study design, outpatient encounters from January-March 2020 (Pre-COVID) were compared to encounters after the pandemic restrictions began April-June 2020 (COVID-Impacted). The primary objective was to compare the proportion of synchronous clinic encounters in the gastroenterology ambulatory space conducted virtually before versus after pandemic restrictions were implemented. Secondary objectives were to determine whether patient, disease, or provider-specific factors were associated with virtual care visits or changed with the implementation of pandemic restrictions. Endoscopic encounters were excluded. Descriptive statistics were used to compare patient and encounter characteristics in the Pre-COVID and COVID-Impacted periods. Multiple logistic regression modeling was used to evaluate the association between patient and provider characteristics and use of virtual care delivery. Unadjusted and adjusted odds ratio with associated 95% CI were estimated.

**Results:**

A total of 4,923 unique patients (60.1% Pre-COVID and 39.9% in the COVID-Impacted period) and 6,659 encounters were identified. The proportion of synchronous clinical visits conducted virtually increased after February 2020, increasing from 25% (Pre-COVID) to 91% (COVID-Impacted). The Pre-COVID versus COVID-Impacted periods also differed with respect to median patient age (56 vs. 59, P = 0.000), mean proximity to the hospital (40km vs. 48km, P = 0.007) and proportion of new consults deemed urgent (9.8% vs. 20.0%, P = 0.000). Patients with family physicians, return visits, and patient age greater than 65 years were associated with the use of synchronous virtual care visits.

**Conclusions:**

This project details the abrupt and significant disruption in in-person ambulatory, non-endoscopic digestive care and the dramatic uptake in virtual care delivery as a result of COVID-19 restrictions in Halifax, NS. Future research will explore virtual care use as pandemic restrictions ease to inform how virtual care is integrated into post-pandemic practice to guide new standards of care.

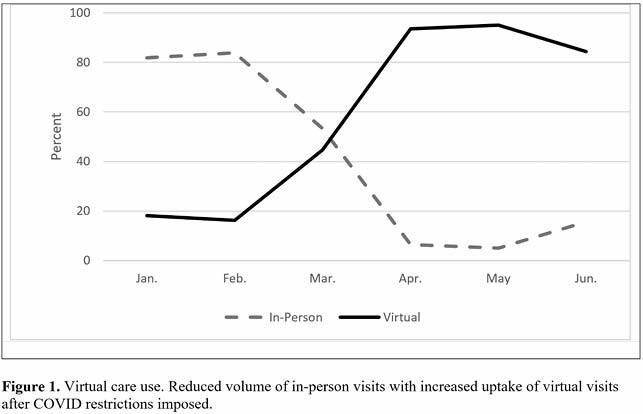

**Funding Agencies:**

None

